# Could it be groove pancreatitis? A frequently misdiagnosed condition with a surgical solution

**DOI:** 10.1111/ans.17939

**Published:** 2022-08-02

**Authors:** Joshua Teo, Arul Suthananthan, Ryan Pereira, Mark Bettington, Kellee Slater

**Affiliations:** ^1^ Hepatopancreatobiliary Surgery Unit, Level 4 The Princess Alexandra Hospital Brisbane Queensland Australia; ^2^ Envoi Pathology Brisbane Queensland Australia; ^3^ General Surgery Greenslopes Private Hospital Brisbane Queensland Australia

**Keywords:** groove, pancreatitis, paraduodenal, pancreaticoduodenectomy, whipples

## Abstract

**Background:**

Groove pancreatitis (GP) is an underrecognised subtype of chronic pancreatitis, focally affecting the area between the duodenum and pancreatic head. It most commonly affects males between 40 and 50 years of age with a history of alcohol misuse. Patients most commonly complain of abdominal pain and vomiting. Due to its focal nature, it is a potentially surgically treatable form of chronic pancreatitis. We report results of patients surgically treated for groove pancreatitis followed by a literature review of patient outcomes post resection.

**Methods:**

A retrospective chart review of patients with histopathologically confirmed GP post‐surgical resection at the Princess Alexandra Hospital and Greenslopes Private Hospital in Brisbane, Australia was conducted between 2013 and 2020. Diagnosis was confirmed histologically when Brunner gland hyperplasia and chronic inflammation/fibrosis were found within the pancreaticoduodenal interface. Preoperative and postoperative symptoms were analysed along with complications. Additionally, a systematic review on outcomes of patients undergoing pancreaticoduodenectomy (PD) for GP was performed from three databases.

**Results:**

Eight patients underwent surgery for GP. Elimination of preoperative symptoms was achieved in five of the eight patients. Major complications included one take back to theatre for pancreatic leak. Our literature review found complete resolution of pain and vomiting in 80% of GP patients after PD.

**Conclusion:**

Optimal management of GP begins with early recognition. Symptoms from GP are likely to respond well to surgical intervention. We advocate for aggressive surgical resection in a patient with a high index of suspicion for GP.

## Graphical abstract

Groove pancreatitis is an underrecognised subtype of chronic pancreatitis that most commonly presents as vomiting and abdominal pain in middle aged alcoholic men. This potentially debilitating condition can be successfully managed with surgery.

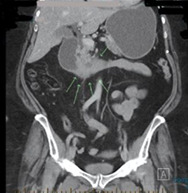

## Introduction

Chronic pancreatitis is a relatively common surgical pathology with an annual incidence of 7–10 per 100, 000. Aetiology is multifactorial and may be a result of environmental (alcohol, nicotine and nutritional), hereditary, autoimmune factors or as a result of pancreatic ductal obstruction by calcification.^1^ Groove pancreatitis (GP) is a subtype of chronic pancreatitis focally affecting the area between the first and second parts of the duodenum and the pancreatic head.^2^ It was first described in 1970 and may account for 3.5%–24.4% of pancreaticoduodenectomy (PD) specimens in patients with symptomatic chronic pancreatitis. However, given it is frequently misdiagnosed or not considered, its true incidence is unknown.^2^, ^3^, ^4^, ^5^

The demographic of patients with GP are predominantly males, aged 40–50 years old, with a history of alcohol misuse. Patients often complain of abdominal pain, vomiting and marked weight loss due to gastric outlet obstruction from duodenal stenosis.^6^, ^7^, ^8^, ^9^, ^10^ Despite the distal bile duct passing through the head of the pancreas, obstructive jaundice is an uncommon presentation.^7^, ^9^, ^11^

Management of this condition lies in the ability to recognize and differentiate it from other forms of chronic pancreatitis, as well as from a pancreatic or peri‐ampullary malignancy which it often mimics. Due to its focal nature, it is a potentially surgically treatable form of chronic pancreatitis with good relief of symptoms. We report the results of eight patients surgically treated for GP followed by a review of the literature of outcomes following PD.

## Methods

Retrospective chart review of patients with histopathologically confirmed GP following surgical resection at the Princess Alexandra Hospital and Greenslopes Private Hospital in Brisbane, Australia was conducted over 7 years (2013–2020). Cases were identified by a search of the hepatobiliary surgeon's logbooks for patients with the discharge diagnosis of GP. Ethics approval was sought and granted by Greenslopes Hospital Ethics Committee as a negligible risk project.

Data collected included gender, alcohol and smoking history, diabetes, history of pancreatitis, presenting symptoms, imaging findings, other investigations, surgical management, post‐operative complications, histopathological findings and long term follow up.

Indications for surgical treatment were, ongoing pain and vomiting in clinically suspected GP recalcitrant to medical and non‐operative measures, or when unable to rule out malignancy. All patients were discussed at a multidisciplinary hepatobiliary meeting where a consensus management decision was formed.

Diagnosis of GP was confirmed histologically when the following features(s) were seen: Brunner gland hyperplasia, chronic inflammation/fibrosis within the pancreaticoduodenal interface, duodenal cystic dystrophy, heterotopy of pancreatic tissue in the duodenum, with absence of a neoplastic processes.

Surgical complications were graded using the Clavien‐Dindo classification system.^12^ All patients have been routinely followed up by the hepatobiliary unit and assessed for resolution of preoperative symptoms, and requirement for exocrine and endocrine pancreatic replacement.

In addition, a systematic review was performed following the Preferred Reporting Items for Systematic Reviews and Meta‐Analyses (PRISMA) guidelines^13^ (Table 1). Three databases (MEDLINE from 1946, PubMed from 1946, and EMBASE from 1949) were searched to 04 July 2019. Search terms included; groove, pancreatitis and paraduodenal. Included studies were case reports and series with histopathologically confirmed GP in patients that underwent PD. In selected studies, the surgical approach, morbidity, mortality and curative outcome of PD were evaluated where follow up duration was reported.

**Table 1:** PRISMA table illustrating method of literature review

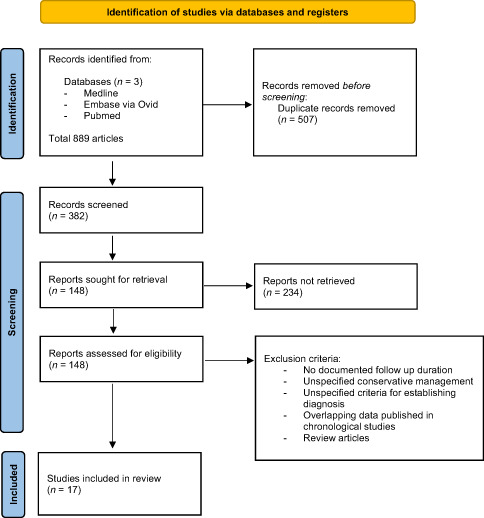

## Results

Eight patients with a clinical diagnosis of GP were identified from review of clinical records. Their demographics are illustrated in Table 2. Two patients were female, one patient was transgender and on testosterone hormonal treatment, deemed to be the cause of his recurrent pancreatitis. Two of the five patients were drinking >4 standard alcohol drinks at per day at presentation, but stopped when the diagnosis of GP was made. The predominant symptom was vomiting, followed by epigastric pain and weight loss; obstructive jaundice was present in two patients. In all patients, the diagnosis of GP was delayed by weeks or months. Some had been labelled as chronic pancreatitis and were being treated with supportive care and some had a period of prolonged investigation in an attempt to exclude malignancy. There was a general lack of recognition of this condition by non‐hepatobiliary clinicians. As a consequence, the patients were in a poor nutritional state at presentation and required enteral or parenteral feeding prior to definitive management.

**Table 2:** Demographics of 8 patients with groove pancreatitis

Mean age	58.5 years
Gender M:F	6:2
History alcohol abuse	5 (62.5%)
Current smoker	5 (62.5%)
Diabetic on presentation	5 (62.5%)
History of recurrent/chronic pancreatitis	7 (87.5%)
Presenting complaint	
Gastric outlet obstruction	7(88%)
Epigastric pain	4 (50%)
Weight loss	4(50%)
Jaundice	2 (25%)

Pre‐operative diagnosis of GP was based on clinical history and diagnostic investigations. Computed tomography scan (CT) was performed in all patients and typical findings included a hypodense sheet of tissue within the pancreaticoduodenal groove region (*n* = 4), peri‐pancreatic head stranding (*n* = 3), duodenal wall thickening (*n* = 4), radiological evidence of gastric outlet obstruction with delayed passage of oral contrast (*n* = 5) (Fig. 2), pancreatic duct dilatation (*n* = 1) and duodenal intramural cystic change (*n* = 1). Endoscopic ultrasound (EUS) was performed in three patients, each demonstrating duodenal narrowing without a mucosal lesion and unable to admit an endoscope. In one patient, cystic lesions in pancreaticoduodenal groove were seen. Diagnostic cytologic specimens were obtained in two patients, one demonstrating spindle cells, one demonstrating cellular atypia.

All eight patients underwent surgical management. Six patients underwent PD and two patients were treated with double bypass with Roux‐en‐Y hepaticojejunostomy and gastrojejunostomy (DB). Interestingly these two patients who were unable to have PD were the only ones who presented with both jaundice and gastric outlet obstruction. One of these patients had a previously abandoned PD for suspected pancreatic head malignancy due to a perilous dissection around the portal vein. The second patient had Child‐Pugh A cirrhosis with no portal hypertension secondary to hepatitis C and poorly controlled diabetes. It was felt that the risk of morbidity and mortality associated with proceeding with a PD in this patient was too high. Both these patients underwent Roux‐en‐Y biliary bypass and a gastroenterostomy to relieve their symptoms and the pancreatic head and duodenum was left insitu.

**Fig. 1:**
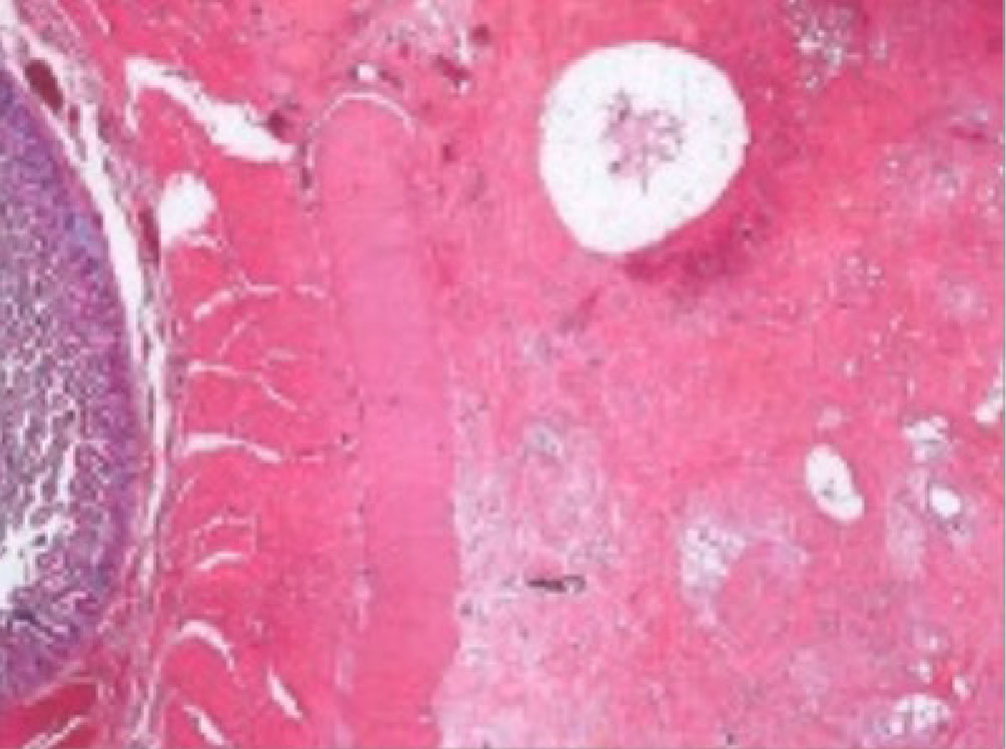
Marked fibrocystic change in the groove between the pancreas and the duodenum associated with acute inflammation and abscess formation. There is florid Brunner gland hyperplasia and duodenitis. Courtesy of envoi pathology.

Histopathological examination confirmed a diagnosis of GP in all six patients who underwent PD. Findings characteristic of GP include Brunner's gland hyperplasia (Fig. 1) (*n* = 6), fibrosis of the pancreaticoduodenal interface (*n* = 6), dilated pancreatic ducts (*n* = 3) and cystic changes in duodenal wall (*n* = 1) (Fig. 3). Dysplasia or malignancy was absent in all cases. The two patients who underwent double bypass have survived >10 years post operatively, meaning that although we were unable to obtain histologic diagnosis, GP was the diagnosis, rather than malignancy.

**Fig. 2:**
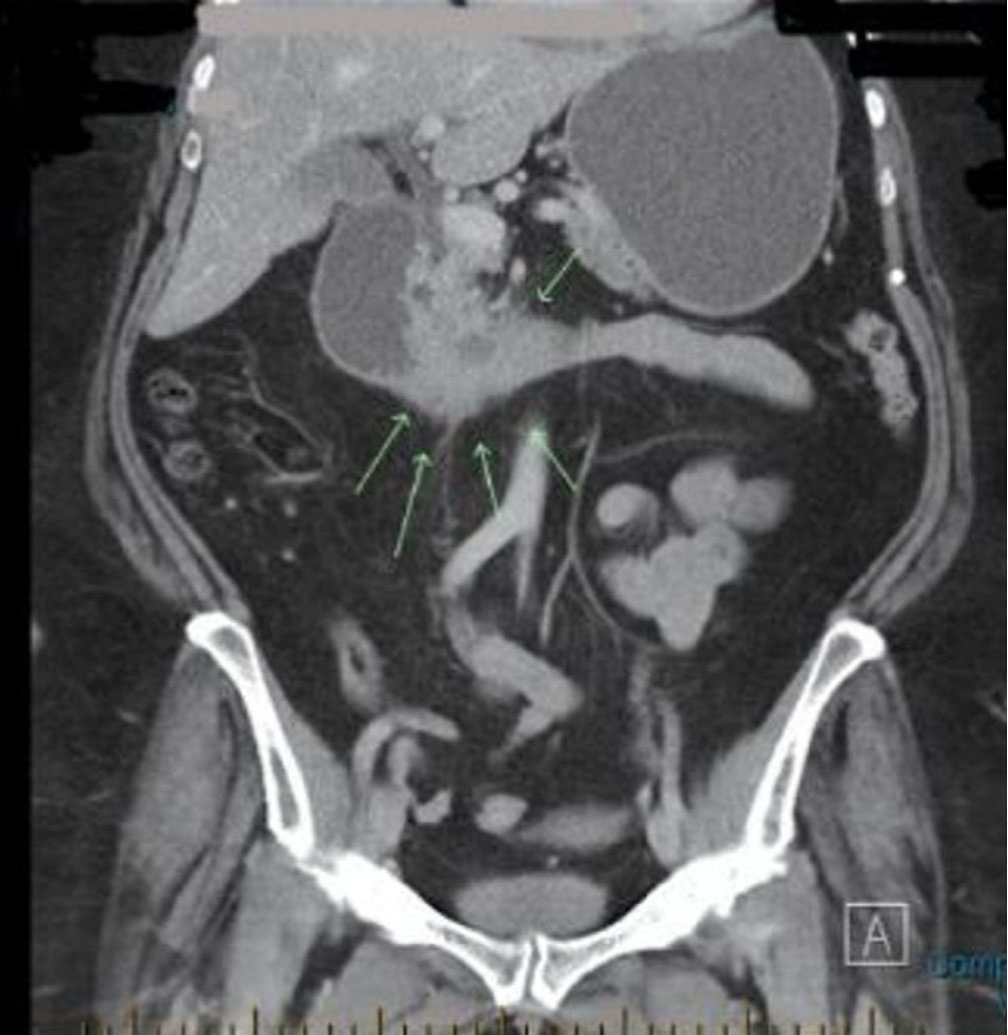
Thickening of the second and third part of the duodenum (arrows) associated with gastric outlet obstruction.

**Fig. 3:**
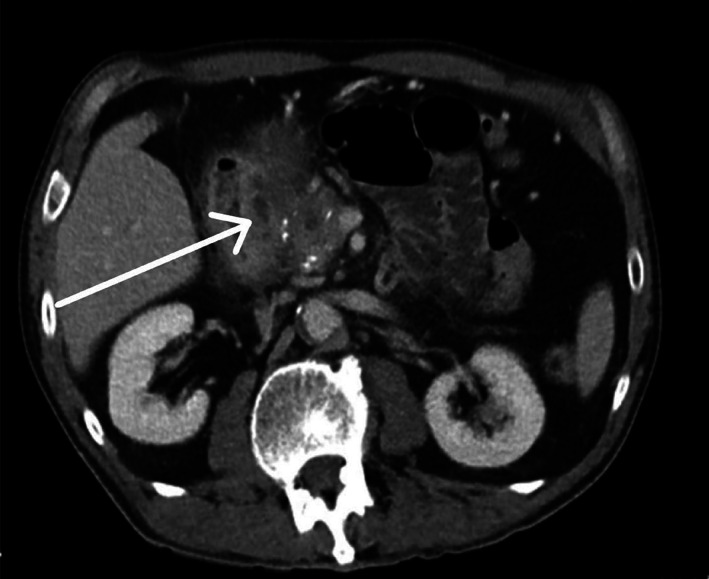
Axial CT image of groove pancreatitis with paraduodenal cysts and chronic calcific changes in the pancreatic head.

There was no post‐operative mortality. One patient suffered post‐operative haemorrhage on day one and a pancreatic leak on day five requiring operative intervention and prolonged drainage. One patient developed a 15 cm pseudocyst 12 months post‐operatively, managed with endoscopic transgastric drainage. One patient developed a stricture at their biliary anastomosis 6 months after PD and was treated by percutaneous transhepatic dilatation.

Follow up period was 6–90 months (median 42 months). Elimination of symptoms (relief of vomiting, weight gain and elimination of pain,) was achieved in five patients following PD; one patient continues to have chronic pain. In the two patients that underwent surgical bypass alone, gastrointestinal obstruction and biliary obstruction was relieved with subsequent weight gain however chronic pain persisted. Three patients did not have pre‐operative diabetes and none of these patients were diabetic following surgery.

No patients required nutritional support beyond the peri‐operative period. All patients were placed on pancrelipase tablets postoperatively to regulate bowel motions and improve nutrition.

## Literature review

The initial search found 889 articles. Key words included groove, pancreatitis, paraduodenal, pancreaduodenectomy, Whipples. Duplicate articles (*n* = 507) were excluded; a further 234 articles were excluded after a title and abstract review. Full text review was performed for 131 articles. Seventeen studies were included for final review, comprising 146 patients with histopathologically confirmed GP.^7^, ^9^, ^11^, ^14^, ^15^, ^16^, ^17^, ^18^, ^19^, ^20^, ^21^, ^22^, ^23^, ^24^, ^25^, ^26^, ^27^ Mean age was 42.1 years; 79% of patients were male.^9^, ^11^, ^15^, ^16^, ^17^, ^18^, ^19^, ^20^, ^22^, ^23^, ^24^, ^25^, ^26^, ^27^ A smoking history was significant in 71% of patients (95% CI: 46%–88%).^9^, ^15^, ^16^, ^19^, ^24^, ^25^, ^26^ Alcohol misuse was present in 75% of patients (95% CI: 60%–86%).^9^, ^11^, ^15^, ^16^, ^19^, ^24^, ^25^, ^26^, ^27^ Most common symptoms were vomiting (60% [95% CI 46%–73%]), abdominal pain (91% [95% CI 81%–96%]) and weight loss (67% [95% CI: 48%–81%]).^9^, ^11^, ^15^, ^16^, ^19^, ^24^, ^25^, ^26^, ^27^ PD was performed in 97.95% of cases (*n* = 143). Post‐operative morbidity was classified using Clavien–Dindo (CD) system.^12^ Major morbidity (CD≥3A) rate was 5.5% (*n* = 8) for all patients, mortality rate was 2% (*n* = 3). Following PD, the rate of clinically significant pancreatic fistulas was 3.5% (*n* = 5) and mortality rate was 2.05% (95% CI: 4.26%–5.89%). Mean follow up was 31.19 months (95% CI: 17.86–44.51%). Complete resolution of pain and obstructive symptoms after PD was achieved for 80.14% (95% CI: 72.73%–86.27%) of patients with at minimum partial resolution for the remainder of patients.

## Discussion

The aetiology of GP remains incompletely understood and is likely to involve both structural and anatomic factors. The final common pathway appears to be related to pancreatic ductal obstruction with the extravasation of activated proteolytic enzymes triggering pancreatitis, resulting in inflammation and fibrosis. One theory of the cause of PD is obstruction of the duct of Santorini. Unlike the main pancreatic duct, the duct of Santorini does not have the sphincter of Oddi to regulate emptying. Partial obstruction of this duct may occur as a result of heterotopic pancreatic tissue and Brunner's gland hyperplasia infiltrating the wall of the duodenum. There is also a strong association between GP and alcohol and tobacco use. Alcohol decreases the volume and increases the viscosity of pancreatic secretions that result in crystal and stone formation that may contribute to pancreatic ductal obstruction.^7^, ^28^

Optimal management of GP begins with its early recognition. Whilst GP is not common, one of the goals of this paper is to draw attention to its existence because it is a type of pancreatitis that is likely to be treated well with surgical intervention. Unless it is recognized, it is possible that the patient may undergo significant suffering through conservative management.

Several specific clinical symptoms and diagnostic signs point towards a diagnosis of GP. There is frequently a history of recurrent or chronic pancreatitis usually associated with alcohol misuse. The thickening of the duodenal groove means that vomiting due to gastric outlet obstruction with corresponding weight loss is a likely presentation. Chronic pain from underlying inflammation is another feature of GP, but not characteristic of it. Jaundice is not common but its presence must always raise suspicion of a malignant process.

In our series of eight patients with histologically confirmed GP, all but one gave a history of previous episodes of pancreatitis, six from alcohol misuse. Seven patients presented with the typical features of gastric outlet obstruction.

With experience, specific radiological signs can reliably point to the diagnosis of GP and Magnetic Resonance Imaging (MRI) in addition to CT and can add value. Radiological features include soft tissue density and widening in the duodenal/pancreatic head interface and cystic change in the duodenal wall. There may be features of chronic pancreatitis such as calcifications in the remaining pancreas. Whilst the pancreatic head may appear bulky there is usually a of lack of an obvious hypodense mass that might suggest malignancy. Pancreatic duct dilatation is a less specific sign.^29^, ^30^, ^31^, ^32^, ^33^, ^34^, ^35^ Vascular encasement is not a feature of GP and its presence should also alert to the possibility of a malignant process.^30^ Boninsegna *et al*. reported a retrospective study of the MRI findings of 28 patients with histologically confirmed GP. The most suggestive signs of GP are signal hyperintensity in the delayed phase, iso‐intensity on diffusion‐weighted imaging (DWI), absence of double duct sign, presence of cysts within the lesion and focal thickening of the duodenum.^6^ Similarly EUS serves as an adjunct to diagnostic imaging, and allows multi‐modality assessment and biopsy, especially when malignancy is suspected. Consistent findings of fine needle aspiration specimens for histopathologically proven GP are stromal spindle cells. Inflammatory cells, duodenal contaminants, and sheets of bland, loosely cohesive epithelial cells may also be seen but do not assist with the pre‐operative diagnosis.^36^, ^37^, ^38^ Endoscopy will also be helpful to exclude other causes of gastric outlet obstruction. GP should be in the differential diagnosis when there is no mucosal lesion present in the duodenum and where the obstruction appears to be as a result of extrinsic compression of the duodenum.

Differentiating GP from malignancy may be difficult and involves correlation of both clinical and imaging features.^9^, ^10^, ^11^, ^13^, ^14^, ^15^, ^29^, ^30^, ^31^, ^32^, ^33^, ^36^, ^37^, ^38^, ^39^, ^40^, ^41^, ^42^, ^43^, ^44^, ^45^ GP tends to occur in younger patients between the 4th and 5th decade of life with a prior history of alcohol misuse and a longer history of symptoms of recurrent or chronic pancreatitis. In direct contrast to malignancy of the head of the pancreas, jaundice is the least common symptom, whilst vomiting is the most frequent. The characteristic findings on CT as described above do not rule out an underlying malignancy. However, the absence of a hypodense mass on CT and EUS provides a degree of reassurance.

Treatment for patients with GP aims to relieve gastric outlet obstruction, correct malnutrition and promote weight gain.^46^ There have been reports of endoscopic modalities to address GP as an alternative to surgery including cystenterostomy, pancreatic or biliary sphincterotomy with stent placement and duodenal dilation.^47^, ^48^, ^49^ Whilst being minimally invasive, these interventions are not a single stage definitive treatment and also fail to rule out the possibility of pancreatic or duodenal malignancy. Chantarojanasiri *et al*. reported seven patients with GP managed endoscopically with pancreatic duct drainage and stenting via the minor papilla. Five cases were able to achieve both clinical and imaging response to treatment. The median total number of sessions was five (3–31), with a median of four sessions (range, 3–5) performed within the first 6 months.^48^ In a series by Casetti *et al*. all three endoscopically treated patients eventually needed definitive therapy with PD.^7^

Given the number of interventions needed to achieve symptom control, an argument for surgical resection balances the logistical and economic benefits for a similar clinical outcome, especially considering the risk of a missed malignancy without a resection. The surgical options include bypass procedures such as gastrojejunostomy with the addition of choledochojejunostomy for biliary involvement, if the patient is unfit for radical resection. These have the disadvantage of no definitive histology and leaving the diseased duodenum and head of pancreas *in‐situ* may not completely relieve the patient's symptoms.

The definitive surgical procedure is PD. This has several advantages. The first is that it will completely remove the affected area. In a study by Casetti *et al*., histopathological examination of the PD specimens in patients with GP often revealed neural proliferation characterized by hyperplastic nerves intimately integrated with proliferating islets.^7^ It is the aim of an en‐bloc resection to remove this diseased hyperplastic neural tissue and this may assist in relieving the chronic pain associated with this condition. Finally, as no imaging or endoscopic assessment is able to rule in or out malignancy, PD allows a definitive diagnosis of benign disease based on the histopathological analysis of the resected specimen.

We advocate for aggressive surgical resection in a patient with a high index of suspicion for GP. Moreover, when in doubt over the possibility of a malignancy, the management decision should lean even further towards surgical resection.

In our systematic review of 17 studies (Table 3) with 146 patients of which 143 (98%) underwent PD, complete resolution of symptoms was achieved in more than 80% of patients whilst the remaining 20% achieved some improvement of their pre‐intervention symptoms over a 31 month follow up.^7^, ^9^, ^11^, ^14^, ^15^, ^16^, ^17^, ^18^, ^19^, ^20^, ^21^, ^22^, ^23^, ^24^, ^25^, ^26^, ^27^ The major morbidity and mortality rates were 5.5% and 2% respectively, with a clinically significant pancreatic fistula rate of 3.5%. This demonstrates that PD can be performed safely, with acceptable outcomes despite a higher technical difficulty. Similarly, in our clinical series of six patients who underwent PD, five achieved complete resolution of pre‐treatment symptoms, with one major complication and no mortality. We found gastroenterostomy and/or hepaticojejunostomy suboptimal for pain control and for some time, there was still the lingering question of underlying malignancy. Given the lasting patency of operative biliary and gastroenteric bypass we would still favour this approach over long‐term stenting in cases of suspected GP who for whatever reason are unable to undergo PD.

**Table 3:** Systematic review ‐ outcome following pancreaticoduodenectomy for groove pancreatitis

Author	Patients (*n*)	Complete resolution pain	Partial improvement pain	Persistent severe pain	Weight gain	Follow up (months)	Peri‐Operative mortality
Chatelain (2005)	2	2	0	0	N/A	12	0
Jouannaud (2006)	12	12	0	0	N/A	12	0
Pessaux (2006)	12	10	0	1		64 (mean)	1
Rahman (2007)	10	9	1	0	Unspecified increase in all	12 (median)	0
Galloro (2008)	1	1	0	0	N/A	14	0
Casetti (2009)	46	35	11	0	BMI increase by 2.7	96.3 (median)	0
Isaacs (2010)	2	2	0	0	Unspecified increase in all	96 (mean)	0
Manzelli (2011)	5	‐	5	0	Unspecified increase in all	12	0
Kim (2011)	5	3	0	0	N/A	32	2
Goransky (2013)	1	1	0	0	N/A	15	0
Latham (2013)	2	2	0	0	N/A	6	0
Rabi (2014)	14	12	0	0	N/A	14.2 (median)	2
Egorov (2014)	29	23	6	0	6 kg (mean)	19	0
Oza (2015)	8	5	2	0	Unspecified increase in 6	11.52 (mean)	1
Desai (2016)	1	1	0	0	N/A	60	0
Sanchez‐Bueno (2016)	8	7	0	1	N/A	>12	0
Lekkerkerker (2016)	8	4	3	0	N/A	24 (median)	1
Total	166	129 (77.7%)	28 (16.9%)	2 (1.2%)	–	–	7 (4.2%)

## Conclusion

Whilst uncommon and underappreciated, GP appears to display a specific set of pathognomonic features and its diagnosis should be considered whenever a patient presents with signs and symptoms of gastric outlet obstruction. This is particularly the case when imaging demonstrates thickening of the duodenopancreatic interface or cystic change in the duodenal wall and there is a history of alcohol misuse and pancreatitis. The differential diagnosis of GP may also include pancreatic adenocarcinoma, duodenal lymphoma, autoimmune pancreatitis, eosinophilic duodenitis and chronic pancreatitis causing an inflammatory duodenal stricture, with every effort made to rule these out. The definitive diagnosis of GP is best established following the histopathological examination of the resected surgical specimen. Our case series whilst small, when combined with the other available literature, seems to support an aggressive surgical approach to GP. This must be balanced against the patient's co‐morbidities and the risks of undergoing a PD.

## Author contributions

**Joshua Teo:** Project administration; visualization; writing – review and editing. **Arul Suthananthan:** Formal analysis; investigation; methodology; writing – original draft. **Ryan Periera:** Data curation; investigation; methodology; writing – original draft. **Mark Bettington:** Resources. **Kellee Slater:** Conceptualization; data curation; formal analysis; investigation; methodology; resources; supervision; writing – original draft; writing – review and editing.

## Conflict of interest

None declared.
